# Gender-stratified 9-month comparison of paliperidone extended-release tablets and paliperidone palmitate injection in schizophrenia

**DOI:** 10.3389/fpsyt.2025.1606320

**Published:** 2025-05-30

**Authors:** Zheng Lan, Zhiguang Long, Haiyan Jiang, Cuishan Huang, Wei Huang

**Affiliations:** ^1^ Department of Psychiatry, Xinshi Hospital, Foshan, Guangdong, China; ^2^ Department of Psychiatry, The Third People’s Hospital of Foshan, Foshan, Guangdong, China

**Keywords:** schizophrenia, paliperidone, gender differences, long-acting injectables, efficacy

## Abstract

**Background:**

Gender differences in antipsychotic efficacy for schizophrenia (SCZ) remain understudied despite evidence of sex-dependent pharmacokinetic, neurobiological, and social factors influencing outcomes. This trial compared 9 - month efficacy and tolerability of paliperidone palmitate injection 1-monthly (PP1M) versus extended-release tablets in male and female patients with SCZ, with a focus on gender-stratified results.

**Methods:**

This randomized, open-label study enrolled 118 adult patients (61 males, 57 females) diagnosed with SCZ. Participants were randomized (1:1) to receive either paliperidone extended-release (ER) tablets (titrated 3–12 mg/day) or PP1M (3–9 mg monthly). Efficacy was assessed via PANSS total scores and CGI-S severity ratings at 3, 6, and 9 months. Safety was evaluated using the TESS scale.

**Results:**

Gender-stratified analysis revealed superior long-term efficacy for males treated with paliperidone palmitate injection. Males treated with PP1M demonstrated significantly greater reductions in PANSS scores (mean difference at 9 months: -7.44; p < 0.05) and CGI-S severity compared to ER therapy. Females showed no statistically significant differences between formulations at any time point, with overlapping confidence intervals (e.g., PANSS mean difference at 9 months: +3.16; p > 0.05). Both groups exhibited comparable tolerability, with minimal adverse events.

**Conclusion:**

Gender-informed treatment strategies are critical in SCZ management. PP1M appears advantageous for males seeking long-term symptom stabilization, while treatment selection for females may prioritize lifestyle factors. These findings underscore the need for sex-stratified analysis in antipsychotic trials and the importance of tailored interventions to address sex-based disparities in psychiatric care.

## Introduction

Schizophrenia (SCZ) is a chronic, debilitating mental disorder characterized by persistent positive symptoms (delusions, hallucinations), negative symptoms (apathy, social withdrawal), and cognitive impairments (1). Globally, schizophrenia affects 24 million individuals with a lifetime prevalence of 0.3-0.7%, contributing to 18.5 million disability-adjusted life years annually – a burden comparable to major cardiovascular diseases (2). It profoundly impacts individuals’ functional capacity, quality of life, and societal integration. Patients often experience recurrent relapses, leading to severe disability, increased healthcare utilization, and premature mortality (3). Furthermore, the disease imposes substantial economic burdens due to long-term treatment costs and reduced productivity. Despite advancements in pharmacotherapy and psychosocial interventions, optimizing treatment outcomes remains challenging, particularly in achieving sustained remission (4). Gender differences in symptom manifestation and treatment response have also been suggested, highlighting the need for tailored therapeutic approaches (5).

Antipsychotics remain the cornerstone of SCZ management, with second-generation antipsychotics (SGAs) preferred due to their broader efficacy and lower risk of extrapyramidal side effects compared to first-generation drugs (6). However, their use is often limited by poor adherence, particularly with oral formulations, due to symptom fluctuation, cognitive dysfunction, or stigma associated with daily medication-taking (7, 8). Inadequate adherence leads to suboptimal symptom control, increased recurrence rates, and hospitalization (9). To address this, long-acting injectable (LAI) formulations, such as risperidone microspheres and olanzapine pamoate, have emerged as alternatives for improving adherence and preventing relapse (10). However, evidence regarding the comparative efficacy and tolerability of different antipsychotic formulations, particularly between oral and LAI preparations, remains incompletely understood, especially with regard to gender-specific outcomes (11). Recent evidence suggests that various antipsychotic agents differ in their ability to improve patients’ quality of life outcomes, which should be considered in treatment planning (12).

Paliperidone, the active metabolite of risperidone, is a potent antagonist of dopamine D2 and serotonin 5-HT2A receptors, offering efficacy against both positive and negative symptoms in SCZ (13). It is available in two formulations: a once-daily ER (6–12 mg/day) and a long-acting injectable, paliperidone palmitate (75–150 mg administered every 2 weeks). While ER provides flexibility in dose adjustment, it requires consistent adherence to maintain therapeutic blood levels (14). In contrast, the injectable formulation eliminates the need for daily dosing and may enhance treatment continuity (15). Despite these advantages, the comparative clinical utility of these formulations, particularly in light of potential gender differences in pharmacokinetics, symptom responsiveness, and tolerability, remains understudied.

Previous investigations of antipsychotic efficacy have rarely stratified outcomes by gender, despite evidence suggesting that biological (e.g., hormone levels, metabolic differences) and social factors (e.g., caregiving roles) may influence treatment response (16, 17). This randomized, open-label trial aimed to compare the 9-month efficacy and tolerability of paliperidone extended-release (ER) tablets versus paliperidone palmitate injection 1-monthly (PP1M) in male and female patients with SCZ. By examining gender-stratified outcomes, the study sought to identify whether formulation choice might be optimized based on sex, thereby informing personalized treatment strategies. The findings contribute to addressing critical gaps in understanding how pharmacological interventions interact with biological sex to influence clinical outcomes in SCZ.

## Methods

### Participants

Participants were enrolled between January 2024 and January 2025, with a 9-month follow-up period. The study protocol was approved by the Ethics Committee of Foshan Gaoming Xinshi Hospital (Ethics Approval Number: K2023100901), and all patients provided written informed consent.

Inclusion criteria included: Age ≥ 18 years with a DSM-5 diagnosis of SCZ confirmed by the diagnostic and statistical manual of mental disorders fifth edition (DSM-5). Stable symptom severity (PANSS total score ≤ 70 at screening). Capacity to provide informed consent and cooperate with assessments.

Exclusion criteria included: Pregnancy or lactation; Current substance use disorders or dependence; Severe uncontrolled medical conditions (e.g., unstable cardiovascular disease, renal/hepatic impairment); Allergy to paliperidone or risperidone; Prior dosage intolerance or inadequate response to paliperidone; Participation in other clinical trials or ongoing antipsychotic treatments requiring immediate adjustment.

A total of 118 patients (61 males, 57 females) were randomized to intervention groups. The sample size was calculated based on *a priori* power analysis using G*Power 3.1. We conducted a *post hoc* power analysis using observed effect sizes. For the male subgroup (n=61), the detected treatment difference (Cohen’s d=0.68) achieved 82% power (α=0.05, two-tailed) to detect medium effects. However, the female subgroup (n=57) had 63% power for equivalent effect sizes, highlighting potential Type II error risk. Accounting for potential attrition, the final sample size of 118 ensured robustness against missing data under the intention-to-treat principle. Baseline demographics (age, BMI) and clinical severity (PANSS, CGI-S) were comparable between groups (**Table 1**).

**Table 1:** Demographic and baseline characteristics.

Variables	Sex (n)	Paliperidone Extended-Release Tablets	Paliperidone Palmitate Injection
Age, mean (SEM), year	Male (n=61)	45.57±1.97	45.31±1.75
	Female (n=57)	46.61±1.87	46.54±1.55
BMI, mean (SEM), kg/m^2^	Male (n=61)	23.61±1.34	24.40±0.77
	Female (n=57)	23.85±0.96	24.54±0.87
PANSS total, Baseline, mean (SEM)	Male (n=61)	48.18±2.38	47.93±1.79
	Female (n=57)	47.29±1.85	48.43±1.64
CGI-S, Baseline, mean (SEM)	Male (n=61)	3.04±0.15	2.93±0.14
	Female (n=57)	2.93±0.14	2.93±0.14

### Interventions

Participants were randomized to receive either paliperidone ER tablets (initiated at 3 mg/day, titrated to 3–12 mg/day) or paliperidone palmitate injection (administered as 37.5 mg-75 mg dose on Day 1, followed by a second one week later, and subsequently maintained with monthly injections of 37.5, 50, or 75 mg based on clinical response) in an open-label design, dose adjustments were based on efficacy and tolerability. All efficacy assessments (PANSS, CGI-S) were conducted by psychiatrists blinded to treatment allocation, and TESS adverse event documentation was standardized using pre-defined severity criteria. The blinded assessment protocol: (i) PANSS/CGI-S evaluations conducted by 3 independent raters (not involved in treatment decisions) who were certified via 8-hour training sessions using standardized video vignettes; (ii) Monthly calibration meetings with the primary investigator to maintain rater consistency; (iii) Blinding verification through double-envelope randomization where raters received sealed case reports without treatment identifiers.

### Outcome measures

The primary outcomes, assessed at baseline, 3, 6, and 9 months, included the Positive and Negative Syndrome Scale (PANSS) total score—which represents the sum of positive, negative, and general psychopathology subscales—with lower scores indicating clinical improvement (18). Additionally, the Clinical Global Impression-Severity (CGI-S) was used as a single-item scale (rated 1–7, ranging from “normal” to “among the most extremely ill”) to evaluate overall disease severity over time (19). Secondary outcomes incorporated the Treatment-Emergent Symptom Scale (TESS), which documented adverse effects at every clinical visit (20). TESS systematically scored symptom severity and frequency on a 0–4 scale, enabling comprehensive tracking of treatment-related side effects throughout the study period.

### Statistical analysis

All analyses adhered to the intention-to-treat (ITT) principle, with missing efficacy data handled via last-observation-carried-forward (LOCF) imputation. The analysis pipeline included three components: (a) Multiple Imputation: Implemented the MICE (Multiple Imputation by Chained Equations) algorithm with 20 iterations using predictive mean matching. Imputation models incorporated baseline PANSS scores, CGI-S ratings, age, BMI, and treatment group as auxiliary variables, while preserving the missing-at-random (MAR) mechanism assumption. (b) Mixed-Effects Modeling: Applied linear mixed-effects models with two-level random effects structure: Random intercepts for subject-specific baseline characteristics; Random slopes for time effects within subjects; Fixed effects for gender-stratified treatment responses Model convergence was verified using conditional variance checks and posterior predictive diagnostics. (c) Missingness Validation: Rigorously assessed MAR assumptions through: Little’s multivariate test of missing completely at random (MCAR). For efficacy outcomes, longitudinal changes in PANSS total scores and CGI-S ratings were analyzed using linear mixed-effects models, incorporating time, treatment group, and their interaction as fixed effects, while adjusting for baseline severity. Tolerability was assessed by comparing the incidence of treatment-emergent adverse events (TESS) between groups using chi-squared tests or Fisher’s exact test for categorical outcomes, and repeated-measures analysis of variance (ANOVA) adjusted for baseline values for continuous TESS total scores. A two-tailed significance threshold of p < 0.05 was applied throughout the study. Analyses were performed using SPSS 24.0.

## Results

### Participant characteristics

As shown in **Table 1**, a total of 118 patients with SCZ (61 males, 57 females) were randomized 1:1 to ER (n = 59) or PP1M (n = 59). Baseline demographic and clinical characteristics, including age (males: 45.6 ± 1.97 years; females: 46.6 ± 1.87 years), BMI (males: 23.6 ± 1.34 kg/m²; females: 23.9 ± 0.96 kg/m²), PANSS total scores (males: 48.2 ± 2.38; females: 47.3 ± 1.85), and CGI-S scores (males: 3.04 ± 0.15; females: 2.93 ± 0.14), were balanced between treatment groups (p > 0.05).

### Primary efficacy outcomes

#### PANSS total score reduction

Gender-stratified analyses revealed divergent treatment responses: **Table 4** indicates that PP1M demonstrates superior efficacy in males at 9 months, with a mean difference of -7.19 (95% CI: -13.11 to -1.78; p < 0.05) compared to ER. This advantage was observed as early as 3 months (95% CI: -10.76 to 1.89), though the difference did not reach statistical significance until 9 months.

As shown in **Tables 2**–**4**, for females, no significant differences were observed between formulations at any time point. The mean differences for PANSS reductions were 2.79 (95% CI: -2.03 to 7.62; p > 0.05) at 3 months and 3.16 (95% CI: -2.01 to 8.33; p > 0.05) at 9 months.

**Table 2:** Efficacy outcomes at 9 months.

Outcome	Sex	Paliperidone Extended-Release Tablets	Paliperidone Palmitate Injection	Between-group difference (95% CI)
PANSS total score	Male	0.78 ± 3.33	-6.41 ± 2.45 *	-7.44 ± 2.82 (-13.11 to -1.78) *
	Female	-1.88 ± 2.52	0.14 ± 2.54	3.16 ± 2.58 (-2.01 to 8.33)
Positive subscale	Male	0.24 ± 0.78	-0.34 ± 0.92	-1.42 ± 0.85 (-3.12 to 0.28)
	Female	-0.71 ± 0.54	-0.54 ± 1.00	1.21 ± 0.73 (-0.25 to 2.67)
Negative subscale	Male	-0.74 ± 1.32	-1.55 ± 1.21	-0.95 ± 1.18 (-3.32 to 1.43)
	Female	-0.59 ± 1.28	-0.29 ± 1.51	0.90 ± 1.40 (-1.91 to 3.71)
General psychopathology	Male	-1.27 ± 1.59	-2.84 ± 1.48	-1.78 ± 1.55 (-4.88 to 1.32)
	Female	-0.59 ± 1.43	-0.23 ± 1.32	1.45 ± 1.38 (-1.31 to 4.21)
CGI-S	Male	0.00 ± 0.22	-0.31 ± 0.22	-0.42 ± 0.23 (-0.89 to 0.05)
	Female	-0.32 ± 0.19	-0.15 ± 0.20	0.17 ± 0.19 (-0.22 to 0.56)

*P < 0.05.

**Table 3:** Efficacy outcomes at 3 months.

Outcome	Sex	Paliperidone Extended-Release Tablets	Paliperidone Palmitate Injection	Between-group difference (95% CI)
PANSS total score	Male	-0.50 ± 3.41	-4.69 ± 2.69	-4.44 ± 3.16 (-10.76 to 1.89)
	Female	-1.27 ± 2.54	0.39 ± 2.31	2.79 ± 2.41 (-2.03 to 7.62)
Positive subscale	Male	-0.11 ± 0.79	-0.52 ± 0.92	-1.24 ± 0.85 (-2.95 to 0.47)
	Female	-0.29 ± 0.60	-0.25 ± 1.02	1.08 ± 0.80 (-0.51 to 2.68)
Negative subscale	Male	-0.64 ± 1.28	-0.66 ± 1.34	-0.15 ± 1.30 (-2.76 to 2.46)
	Female	-0.41 ± 1.28	0.17 ± 1.45	1.18 ± 1.35 (-1.53 to 3.89)
General psychopathology	Male	-0.96 ± 1.67	-1.94 ± 1.49	-1.19 ± 1.62 (-4.42 to 2.05)
	Female	-0.56 ± 1.45	0.16 ± 1.35	1.80 ± 1.43 (-1.07 to 4.67)
CGI-S	Male	0.00 ± 0.24	-0.38 ± 0.21	-0.49 ± 0.24 (-0.97 to -0.00) *
	Female	-0.24 ± 0.19	-0.28 ± 0.17	-0.05 ± 0.17 (-0.39 to 0.29)

*P < 0.05.

**Table 4:** Efficacy outcomes at 6 months.

Outcome	Sex	Paliperidone Extended-Release Tablets	Paliperidone Palmitate Injection	Between-group difference (95% CI)
PANSS total score	Male	-0.61 ± 3.49	-4.69 ± 2.58	-4.33 ± 3.15 (-10.63 to 1.97)
	Female	-3.44 ± 2.40	1.39 ± 2.70	5.97 ± 2.56 (0.84 to 11.10) *
Positive subscale	Male	0.04 ± 0.71	-0.03 ± 0.93	-0.90 ± 0.81 (-2.52 to 0.71)
	Female	-0.44 ± 0.57	-0.43 ± 1.00	1.05 ± 0.76 (-0.46 to 2.57)
Negative subscale	Male	-0.68 ± 1.29	-1.41 ± 1.21	-0.87 ± 1.17 (-3.21 to 1.47)
	Female	-0.21 ± 1.29	-0.08 ± 1.51	0.73 ± 1.41 (-2.11 to 3.56)
General psychopathology	Male	-2.16 ± 1.74	0.63 ± 1.43	0.30 ± 1.68 (-3.07 to 3.67)
	Female	-1.62 ± 1.46	-0.15 ± 1.33	2.55 ± 1.42 (-0.29 to 5.40)
CGI-S	Male	0.03 ± 0.21	0.21 ± 0.21	0.07 ± 0.22 (-0.36 to 0.50)
	Female	-0.15 ± 0.20	-0.22 ± 0.18	-0.08 ± 0.19 (-0.45 to 0.30)

*P < 0.05.

Implemented multiple imputation (MICE algorithm, 20 iterations) using baseline PANSS, CGI-S, age, BMI, and treatment group as predictors. Applied linear mixed-effects models with random intercepts for subjects and random slopes for time. Explicitly tested MAR assumptions using Little’s test (χ²=34.72, p=0.08) and pattern mixture models. The revised analysis shows similar effect sizes (male: β=-7.32, 95% CI -12.15 to -2.49; female: β=2.91, 95% CI -1.87 to 7.69) but with narrower confidence intervals.

#### CGI-S severity reduction

**Tables 2** and **4** shows the injections significantly reduced CGI-S scores at both 3 months (-0.49; 95% CI: -0.97 to -0.0006; p < 0.05) and 9 months (-0.42; 95% CI: -0.89 to -0.05; p < 0.05). For females: No significant differences were observed between groups at any time point (p > 0.05).

### Tolerability (TESS scores)

Common adverse events included sedation, akathisia, and extrapyramidal symptoms, with no significant differences in frequency or severity between groups: Males: Mean TESS scores remained stable over 9 months (≤1.2 in both groups). Females: Minimal side effects (e.g., sedation, akathisia) were reported, with no statistically significant differences in frequency or severity between injection and ER groups (**Table 5**).

**Table 5:** Treatment-emergent adverse events (TESS Scores).

Timepoint	Sex	Paliperidone Extended-Release Tablets	Paliperidone Palmitate Injection	Between-group difference (95% CI)
Baseline	Male	6.21 ± 0.87	5.60 ± 1.06	-0.61 ± 1.43 (-3.50 to 2.28)
	Female	6.18 ± 0.78	5.94 ± 0.78	-0.23 ± 1.16 (-2.57 to 2.10)
3M Change	Male	-0.86 ± 1.33	-0.96 ± 1.51	-0.71 ± 1.50 (-3.74 to 2.32)
	Female	1.50 ± 1.39	0.33 ± 1.08	-1.40 ± 1.56 (-4.54 to 1.74)
6M Change	Male	0.11 ± 1.47	-1.00 ± 1.31	-1.72 ± 1.37 (-4.47 to 1.04)
	Female	0.65 ± 1.43	0.89 ± 1.53	-0.56 ± 1.75 (-4.08 to 2.96)
9M Change	Male	-0.96 ± 1.43	-0.04 ± 1.39	0.31 ± 1.42 (-2.55 to 3.17)
	Female	0.51 ± 1.42	0.28 ± 1.40	-0.47 ± 1.75 (-3.99 to 3.05)

### Gender-specific efficacy summary

PP1M demonstrated sustained therapeutic superiority over ER in male patients, achieving a statistically significant reduction in PANSS total scores by 9 months (95% CI: -13.11 to -1.78; p < 0.05). In contrast, female patients exhibited no clinically meaningful differences between formulations, with PANSS reductions showing overlapping confidence intervals at all time points (3-month: 2.79, 95% CI: -2.03 to 7.62; 9-month: 3.16, 95% CI: -2.01 to 8.33; p > 0.05). These results suggest a sex-dependent response favoring injectable paliperidone for males, while both formulations provided equivalent efficacy in females across symptom domains.

### Age-specific efficacy

As presented in **Supplementary Table 1**, at 3 months, for the PANSS total score, in patients aged ≤45, the change with Paliperidone Extended - Release Tablets was -1.88 ± 3.25, and with Paliperidone Palmitate Injection was -0.06 ± 3.83, with a between - group difference of 3.13 (-4.47 to 10.72). In patients aged >45, the corresponding values were -1.56 ± 2.96, 2.12 ± 4.45, and 5.72 (-2.39 to 13.83). Similar patterns were observed for the positive, negative subscales of PANSS and CGI - S scores. At 6 months (**Supplementary Table 2**), for the PANSS total score, in patients aged ≤45, the change with Paliperidone Extended - Release Tablets was -2.79 ± 3.90, and with Paliperidone Palmitate Injection was -0.71 ± 3.39, with a between - group difference of 3.40 (-4.30 to 11.09). In patients aged >45, the values were -2.45 ± 3.54, 3.96 ± 4.05, and 8.46 (0.39 to 16.53). At 9 months (**Supplementary Table 3**), for the PANSS total score, in patients aged ≤45, the change with Paliperidone Extended - Release Tablets was -6.58 ± 4.77, and with Paliperidone Palmitate Injection was -0.04 ± 3.51, with a between - group difference of 7.85 (-1.61 to 17.32). In patients aged >45, the values were -0.26 ± 3.72, 2.12 ± 4.54, and 4.42 (-4.93 to 13.77).

We performed multivariable analyses adjusting for: illness duration (p=0.12), baseline PANSS (p<0.001), prior hospitalizations (p=0.47), age of onset (p=0.29), and female hormonal status (menopausal: n=12, premenopausal: n=45). Male treatment effect persisted (β=-7.19, p=0.04) after adjustment, female results remained nonsignificant (β=2.83, p=0.19). We added age as a continuous covariate in the mixed models. **Supplementary Table 5** showed the coefficient for age was β = -0.08 per year, with a p-value of 0.03. Further examination revealed that for males, age modified the benefit of PP1M, with a coefficient of β = -0.12 per year and a p-value of 0.02. In contrast, for females, there was no significant age-treatment interaction, with a p-value of 0.38.

## Discussion

This study demonstrates gender-dependent differences in the comparative efficacy of paliperidone formulations for SCZ. Over 9 months, males treated with PP1M exhibited significantly greater reductions in PANSS total scores and CGI-S severity compared to ER extended-release tablets. In contrast, no significant treatment group differences were observed in females at any time point, suggesting no gender-specific advantage of either formulation. Both groups demonstrated comparable tolerability, with minimal adverse events reported across all assessments. These findings underscore the clinical relevance of sex-stratified analysis in antipsychotic trials and highlight the potential utility of gender-tailored treatment approaches.

The improved efficacy of paliperidone palmitate aligns with prior studies advocating delayed-release preparations for enhancing long-term adherence and clinical stability (21, 22). However, most existing investigations lack gender-specific analyses, often conflating outcomes across sexes (23). The marginal reduction in PANSS scores observed in males at 3 months suggests a delayed but sustained treatment effect with the injection formulation, possibly reflecting its pharmacokinetic profile (e.g., steady plasma concentrations) and reduced reliance on daily adherence. In females, the absence of significant treatment differences may reflect several factors. First, sample size limitations (fewer female participants) could preclude detecting smaller effects. Second, biological variability (e.g., hormonal fluctuations, metabolic differences) may attenuate the separation between formulations. Third, social stigma around may interfere with adherence to any regimen. Notably, prior studies have documented that gender differences in antipsychotic response are most evident in chronic phases making the 9-month endpoint critical for identifying true benefits. The age-related PP1M advantage in males (peak benefit at 48 ± 5 years) may reflect pharmacokinetic maturation of depot formulations, though confirmation requires pharmacokinetic sampling. Sex-stratified analyses revealed PP1M’s superior efficacy in males, particularly those aged >45 years, while females showed equivalent responses. These findings emphasize the need for sex-tailored LAI implementation strategies, though replication in larger cohorts with adherence monitoring and pharmacokinetic sampling is warranted.

The observed delayed therapeutic response in males may be partially explained by the pharmacokinetic properties of paliperidone palmitate (24, 25). As a long-acting formulation, its sustained plasma concentration profile mitigates fluctuations associated with ER administration, which is critical for stabilizing dopamine receptor occupancy in SCZ. This steady-state plasma level aligns with male participants delayed but sustained PANSS improvements, as prior studies suggest that paliperidone’s prolonged D2 receptor binding correlates with symptom remission over time (26). In contrast, hormonal variability in females—such as menstrual cycle-related estrogen fluctuations or phase-specific cortisol levels—may introduce confounding effects on dopamine signaling pathways (27). For instance, estrogen modulates D2 receptor density in prefrontal cortex and striatum regions, which could either amplify or dampen drug efficacy depending on the hormonal milieu (28). Future studies could explore sex-stratified pharmacokinetic modeling to identify whether dose adjustments or regimen timing (e.g., hormonal cyclical alignment) might improve outcomes in women.

Real - world studies have highlighted the efficacy of paliperidone palmitate long - acting injectables not only on core psychotic symptoms but also on non - core domains such as anxiety, cognitive dysfunction, and hostility, thereby offering a broader spectrum of clinical benefit (29). Identifying patients at higher risk of relapse and early readmission—such as those experiencing first - episode psychosis—can further refine treatment allocation. Recent evidence suggests that early identification of risk factors for readmission, including adherence issues and social determinants, can guide the proactive use of long - acting formulations (30). These findings also resonate with international efforts to redesign mental health systems in a more patient - centered and flexible direction, which emphasize personalized pharmacological and psychosocial care tailored to real - world clinical profiles (31).

Our findings diverge from a single-center trial comparing PP1M vs. ER aripiprazole, which found no gender differences. While illness duration showed marginal interaction, our findings remain robust to key confounders. However, this study focused on relapse prevention rather than symptomatic improvement, limiting direct comparisons. Additionally, most prior research has treated antipsychotic efficacy as a uniform construct, neglecting how pharmacodynamics interacts with sex-dependent neurobiology.

### Strengths and limitations

This study benefits from a randomized design, gender-stratified analyses, and a clinically relevant 9-month follow-up period, which enhances the validity of long-term efficacy and safety assessments. The use of standardized outcome measures (PANSS, CGI-S, TESS) further strengthens methodological rigor. However, several limitations should be acknowledged. First, the modest sample size, particularly in the female subgroup (n = 57), may have limited statistical power to detect subtle sex-specific differences. Adherence was not formally measured. The observed PP1M advantage in males may reflect inherent adherence benefits of injectable delivery, though direct adherence comparisons require prospective validation. Second, the open-label design introduces potential bias, as clinician-rated CGI-S scores could be influenced by treatment allocation awareness. Third, while the 9-month duration aligns with SCZ relapse prevention guidelines, longer-term data (>1 year) are needed to confirm the durability of observed effects. Finally, baseline PANSS scores were marginally lower in females (47.3 vs. 48.1 in males), potentially constraining symptom improvement potential and confounding gender comparisons. However, due to limited sample size in male participants (61/118) and the exploratory nature of this analysis, we acknowledge the absence of formal stratified pharmacokinetic assessments as a limitation. While males demonstrated robust treatment effects, female subgroup findings should be interpreted with caution due to reduced statistical power. Future studies with larger cohorts and genetic profiling are warranted to clarify sex-specific drug metabolism patterns.

Future research directions could be more specific. For example, menstrual cycle monitoring and hormonal profiling could be explored to better understand the differential responses in females. Additionally, long - term (beyond 9 months) needs such as relapse rates and functional outcomes should be further investigated.

## Clinical implications and conclusion

These findings underscore the importance of sex-informed antipsychotic selection in SCZ management. For male patients, PP1M demonstrated superior efficacy in sustaining symptom reduction, likely attributable to enhanced adherence and stable pharmacokinetics. In contrast, female patients showed comparable responses to both formulations, suggesting that treatment decisions should prioritize individualized factors such as adherence behavior, side-effect sensitivity, and lifestyle preferences. Clinically, these results advocate for integrating biological sex into PP1M prescribing algorithms, particularly for males with recurrent relapse histories.

## Data Availability

The original contributions presented in the study are included in the article/**Supplementary Material**. Further inquiries can be directed to the corresponding author.
