# Supplementary material for: Gender-stratified 9-month comparison of paliperidone extended-release tablets and paliperidone palmitate injection in schizophrenia
Source: Front Psychiatry. 2025 May 30;16:1606320. doi: 10.3389/fpsyt.2025.1606320 (PMC12163612; doi:10.3389/fpsyt.2025.1606320)
Supplement: Supplementary file 1 [file Table1.docx]

Supplemental Table 1. Efficacy Outcomes at 3 Months.

| Outcome | Age | Paliperidone Extended-Release Tablets | Paliperidone Palmitate Injection | Between-group difference (95% CI) |
| --- | --- | --- | --- | --- |
| PANSS | ≤45 | -1.88 ± 3.25 | -0.06 ± 3.83 | 3.13 (-4.47 to 10.72) |
|  | >45 | -1.56 ± 2.96 | 2.12 ± 4.45 | 5.72 (-2.39 to 13.83) |
| Positive subscale | ≤45 | -0.18 ± 1.26 | -1.25 ± 1.01 | -2.07 (-4.27 to 0.13) |
|  | >45 | -0.24 ± 0.66 | 0.45 ± 0.92 | 1.12 (-0.52 to 2.77) |
| Negative subscale | ≤45 | -0.29 ± 1.40 | -0.34 ± 1.64 | 1.51 (-1.53 to 4.56) |
|  | >45 | -0.73 ± 1.23 | -2.16 ± 1.42 | -0.57 (-2.90 to 1.75) |
| General psychopathology | ≤45 | -0.64 ± 1.75 | -1.54 ± 1.64 | -0.29 (-3.37 to 2.80) |
|  | >45 | -1.03 ± 1.62 | -0.71 ± 1.33 | -0.90 (-4.14 to 2.33) |
| CGI-S | ≤45 | -0.04 ± 0.26 | -0.32 ± 0.22 | -0.32 (-0.81 to 0.18) |
|  | >45 | 0.06 ± 0.22 | -0.27 ± 0.17 | -0.34 (-0.71 to 0.03) |

Supplemental Table 2. Efficacy Outcomes at 6 Months.

| Outcome | Age | Paliperidone Extended-Release Tablets | Paliperidone Palmitate Injection | Between-group difference (95% CI) |
| --- | --- | --- | --- | --- |
| PANSS | ≤45 | -2.79 ± 3.90 | -0.71 ± 3.39 | 3.40 (-4.30 to 11.09) |
|  | >45 | -2.45 ± 3.54 | 3.96 ± 4.05 | 8.46 (0.39 to 16.53) |
| Positive subscale | ≤45 | -0.46 ± 1.24 | -0.21 ± 1.11 | -0.75 (-3.10 to 1.60) |
|  | >45 | -0.03 ± 0.63 | -0.24 ± 0.81 | 0.22 (-1.15 to 1.59) |
| Negative subscale | ≤45 | 0.07 ± 1.42 | -0.46 ± 1.69 | 1.04 (-2.19 to 4.26) |
|  | >45 | -0.85 ± 1.22 | -1.21 ± 1.50 | 0.50 (-2.00 to 3.00) |
| General psychopathology | ≤45 | -1.50 ± 1.76 | -0.89 ± 1.85 | 1.21 (-2.33 to 4.76) |
|  | >45 | -2.49 ± 1.63 | -0.41 ± 1.18 | 0.85 (-2.16 to 3.85) |
| CGI-S | ≤45 | -0.07 ± 0.25 | -0.29 ± 0.24 | -0.25 (-0.75 to 0.25) |
|  | >45 | 0.44 ± 0.35 | -0.19 ± 0.17 | -0.65 (-1.36 to 0.06) |

Supplemental Table 3. Efficacy Outcomes at 9 Months.

| Outcome | Age | Paliperidone Extended-Release Tablets | Paliperidone Palmitate Injection | Between-group difference (95% CI) |
| --- | --- | --- | --- | --- |
| PANSS | ≤45 | -6.58 ± 4.77 | -0.04 ± 3.51 | 7.85 (-1.61 to 17.32) |
|  | >45 | -0.26 ± 3.72 | 2.12 ± 4.54 | 4.42 (-4.93 to 13.77) |
| Positive subscale | ≤45 | -0.77 ± 1.23 | -0.25 ± 1.12 | -0.48 (-2.82 to 1.85) |
|  | >45 | 0.01 ± 0.66 | -0.62 ± 0.78 | -0.20 (-1.56 to 1.16) |
| Negative subscale | ≤45 | -0.06 ± 1.47 | -0.89 ± 1.66 | 0.74 (-2.51 to 3.99) |
|  | >45 | -1.17 ± 1.20 | -1.21 ± 1.50 | 0.83 (-1.64 to 3.29) |
| General psychopathology | ≤45 | -0.96 ± 1.69 | -1.43 ± 1.83 | 0.14 (-3.21 to 3.50) |
|  | >45 | -0.86 ± 1.55 | -1.28 ± 1.19 | -1.64 (-4.43 to 1.15) |
| CGI-S | ≤45 | -0.17 ± 0.25 | -0.22 ± 0.24 | -0.09 (-0.61 to 0.43) |
|  | >45 | 0.06 ± 0.22 | -0.17 ± 0.19 | -0.24 (-0.64 to 0.16) |

Supplemental Table 4. Adjusted Treatment Effects by Sex with Key Confounders

| Variable | Male Subgroup | Female Subgroup | Omnibus p-value |
| --- | --- | --- | --- |
| Treatment Effect | β = -7.19 (95% CI -12.15 to -2.49) | β = 2.83 (95% CI -1.87 to 7.69) | N/A |
| Confounders (p-values) |  |  |  |
| Illness duration (months) | 0.12 | 0.29 | 0.15 |
| Baseline PANSS | <0.001 | 0.47 | <0.001 |
| Prior hospitalizations | 0.47 | 0.12 | 0.08 |
| Age of onset (years) | 0.29 | 0.19 | 0.35 |
| Hormonal status | - | Menopausal (n=12) | 0.03 |
|  |  | Premenopausal (n=45) |  |

Supplemental Table 5. Age-Adjusted Treatment Effects with Pharmacokinetic Considerations

| Component | Statistical Result | 95% CI | Subgroup Analysis |
| --- | --- | --- | --- |
| Main Effects |  |  |  |
| Age (continuous) | β = -0.08/year (p=0.03) | -0.14 to -0.02 | - |
| Treatment × Age | β = -0.12/year (p=0.02) | -0.21 to -0.03 | Males only |
| Interaction Effects |  |  |  |
| Age × Sex × Treatment | p=0.11 (trend) | - | - |
| Age × Treatment (Males) | β = -0.12/year (p=0.02) | -0.21 to -0.03 | Peak benefit at 48±5 years |
| Age × Treatment (Females) | β = -0.03/year (p=0.38) | -0.10 to 0.04 | No significant trend |
